# Extranodal lymphoma of the tongue, a very rare entity-report of two cases with literature review

**DOI:** 10.1016/j.ijscr.2018.09.055

**Published:** 2018-11-30

**Authors:** J. Clarke, S. Medford, S. Islam, C. Ramsingh, M. Christopher

**Affiliations:** a Department of Otorhinolaryngology and Head and Neck Surgery, San Fernando Teaching Hospital, Trinidad and Tobago; b Department of General Surgery, San Fernando Teaching Hospital, Trinidad and Tobago; c Department of Clinical Surgical Science, University of the West Indies, St. Augustine, Trinidad and Tobago

**Keywords:** Base of tongue lymphoma, High grade T cell lymphoma, DBCL, Extranodal lymphoma, Waldeyer’s ring

## Abstract

**Background:**

Lymphomas are malignant neoplasms of the lymphocyte cell lines affecting the lymph nodes, spleen and other nonhemopoietic tissues. Of the extranodal lymphomas found in the head and neck region, 3–5% of malignant lymphomas arise in the oral and paraoral region, mainly from Waldeyer's ring. The involvement of the base of the tongue is extremely rare.

**Summarised Case:**

Case 1: 64 year old female who presented initially with an enlarged occipital lymph node which gradually became generalized cervical lymphadenopathy with initial histology confirmed reactive lymphoid hyperplasia. Biopsy of left postero-lateral tongue lesion eventually showed high grade T cell lymphoma.

Case 2: 85 year old male presented with history of dysphagia for one year who was found to have a lesion extending from his base of tongue into the nasopharynx. Histology showed a diffuse B cell lymphoma.

**Discussion:**

Both patients were noted to have lesion of the tongue, but tongue lesions are noted in the literature to be extremely rare. When tongue lymphomas do occur, most are of B-cell origin; the diffuse large-cell variety is the most common. Extranodal lymphomas of the T cell phenotype tend more to be sinonasal in origin than of the tongue, with T cell lymphomas of the tongue being even rarer than B cell lymphomas.

**Conclusion:**

With regards to tumours arising in the tongue, squamous cell carcinomas are still classified as the most common. Lymphomas however, should still be kept in consideration as a differential diagnosis with regards to lesions arising from this site.

## Highlights

• The extranodal lymphoma of the base of the tongue is extremely rare.
• Among the lymphoma of the tongue; B cell lymphoma is more common than T cell lymphoma.
• It should be included in the differentials in patients with tumors arising from this site.
• Early detection and treatment can often result in a complete cure and a better long-term survival.

## 1 Introduction

Lymphomas are malignant neoplasms of the lymphocyte cell lines affecting the lymph nodes, spleen and other nonhemopoietic tissues. They are classified as either Hodgkin's or non-Hodgkin's lymphoma (NHL), either B-cell or T-cell based on their cell of origin. Extranodal lymphomas are a rare presentation [[1], [2], [3]] but tend to occur most commonly in the gastrointestinal tract followed by the head and neck region.

Of the extranodal lymphomas found in the head and neck region, 3–5% [1,2,4] of malignant lymphomas arise in the oral and paraoral region, mainly from Waldeyer's ring. The involvement of the base of the tongue is extremely rare.

We present to you two cases of NHL with involvement of the tongue. To our present knowledge, this is the first reported cases of tongue lymphoma from a Caribbean institution. The management of this two cases as well as literature search was performed and the research work has been reported in line with the PROCESS criteria [5].

## 2 Case 1

This case report is of a 64 - year old female who presented to our institution with complaints of an enlarged swelling in the occipital region. The patient denied any symptoms of night sweats, fever, weight loss or any other swellings to other regions of her body. On examination she was noted to have a 1.5 × 2 cm node in the occipital region which was non-tender, non-erythematous and mobile. The patient had a full ENT examination done which also consisted of a flexible laryngoscopy, all of which proved normal. She was booked for excision of the node. The histology at this time revealed a lymph node with mild reactive features. She was discharged from our clinic at that time but returned with bilateral cervical lymphadenopathy which was again biopsied and showed reactive features.

**Fig. 1:**
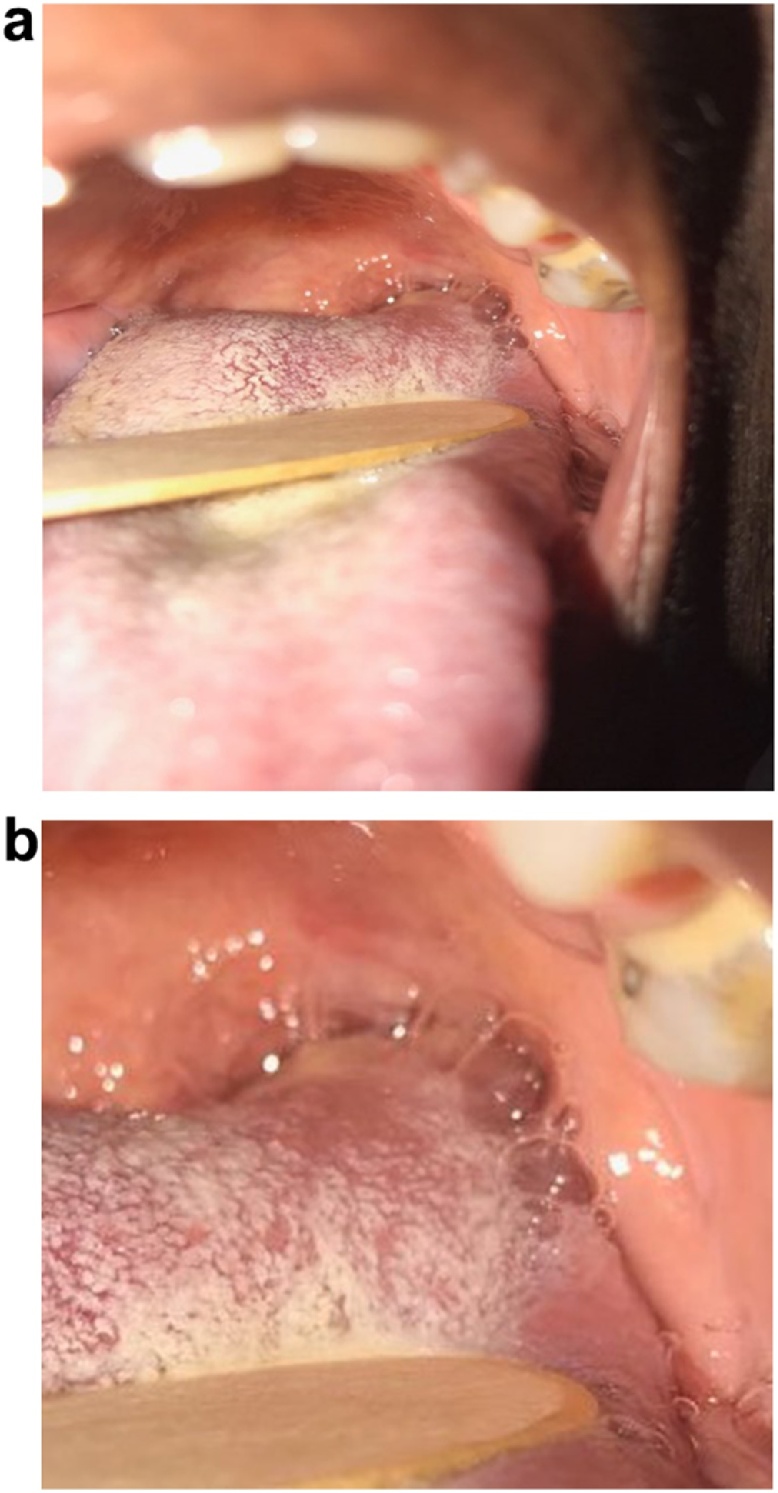
(a, b): showing views of the tongue with the lesion in the left postero-lateral region (as indicated by the black arrows.

Our patient was seen on several occasions and investigated extensively, but all tests were inconclusive. During one of these visits she was noted to have a lesion present on the left postero-lateral part of the anterior tongue, which was palpable below the mucosa but barely visible with the naked eye (Fig. 1a, b).

**Fig. 2:**
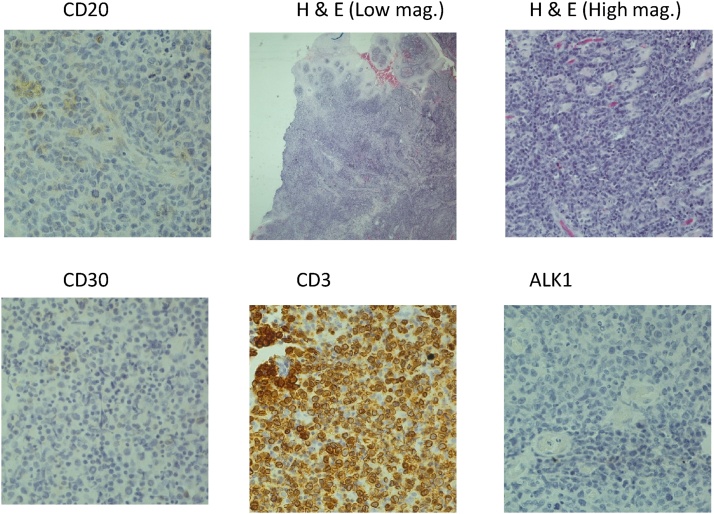
Showing histographs taken of the tongue biopsy and cervical lymph node.

A biopsy was arranged of the left postero-lateral tongue and a left cervical lymph node biopsy. Immunohistochemistry was performed which showed a high-grade T Cell Lymphoma (Fig. 2). The patient was then referred to Haematology for further management of her condition.

## 3 Case 2

**Fig. 3:**
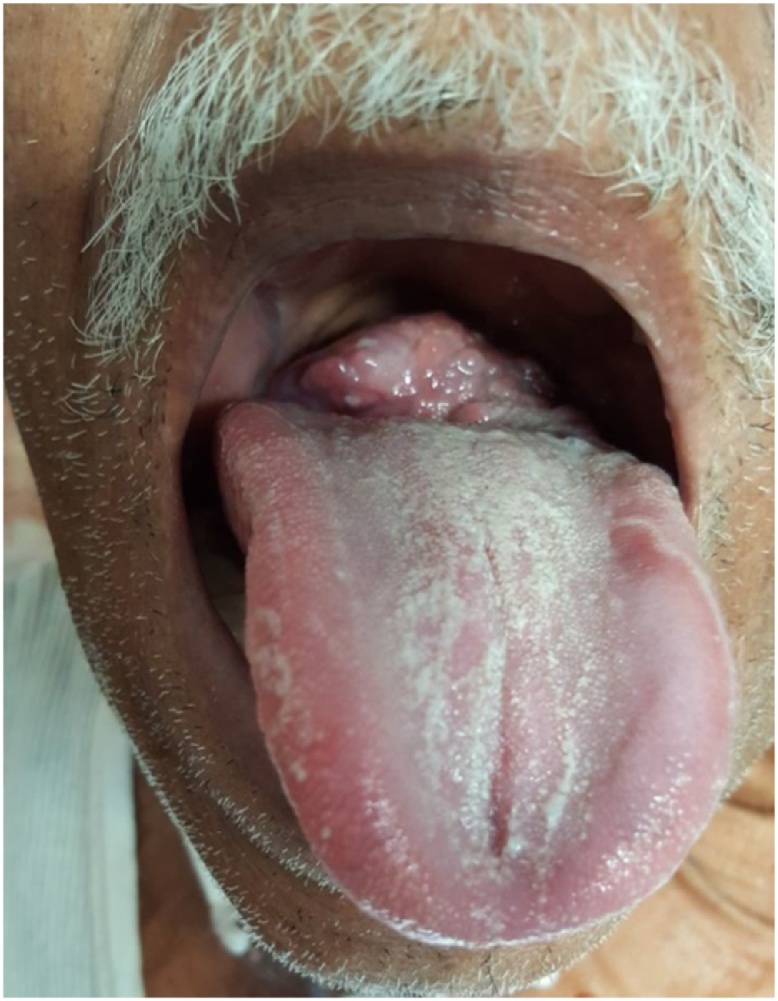
Showing the mass at the base of tongue as indicated by the black arrow.

This second case is of an 85 year old man who presented with a one-year complaint of dysphagia to solids which had been progressively getting worse. He had a sensation of a “lump in his throat”, but no complaints of weight loss, night sweats or fever. The patient was a heavy smoker with a 65 - pack year history. The patient had an examination of his ENT system which revealed a large mass in the base of his tongue which appeared quite vascular (Fig. 3). The oropharynx could not be visualised through the mouth.

**Fig. 4:**
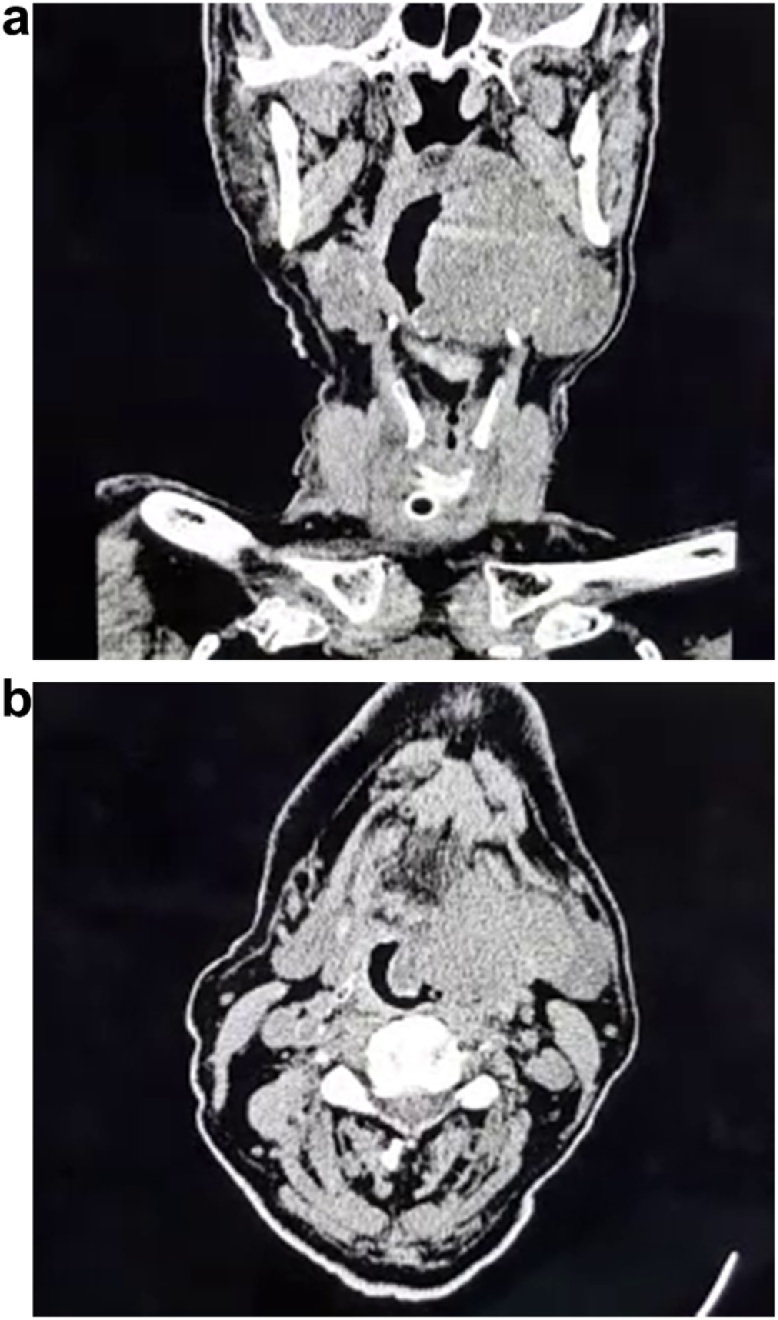
(a) CT scan of the face and Neck (Coronal view); (b): CT scan of the face and Neck (Axial view)- showing 7.4 cm x 6.9 cm x 4.6 cm solid mass at the base of the tongue as denoted by the white arrows.

A flexible laryngoscopy was then attempted which showed the mass extending from the level of the oropharynx down to the base of the tongue, with poor visualisation of the vocal cords. Arrangements were subsequently made for an urgent CT scan of his Head, Neck and Chest, which showed a large, irregular, solid lesion to the left side of the base of the tongue. It measured 7.4 cm (CC) × 6.9 cm (TS) × 4.6 cm (AP) and demonstrated no calcifications or internal cystic component (Fig. 4a, b).

A tracheostomy was then performed under local anaesthesia and biopsies taken from the mass after the patient was placed under general anaesthesia. Immunohistochemistry was done on the specimen which showed normal tonsillar lymphoid architecture effaced by a diffuse population of atypical lymphoid cells with a centroblastic appearance. The cells had a diffuse strong expression of both CD 10 and Bcl-2 and expressed the B-cell marker CD 20 but were negative for CD 3. A diagnosis of diffuse large B-Cell Lymphoma (DLBCL) was thus made. The patient was then referred to Haematology where he underwent treatment of his condition.

## 4 Discussion

Extra nodal Non-Hodgkin Lymphomas (NHLs) are a group of malignancies that involve organs that usually do not contain lymphoid cells. The incidence of NHLs tends to increase with age, which differs to that of Hodgkin lymphoma which has a bimodal distribution. NHLs occurs more in patients with a history of radiation exposure, immunosuppression, congenital immunodeficiency, acquired immune deficiency syndrome (AIDS), rheumatoid arthritis, celiac disease, and Sjögren syndrome [4,6]. Also, the risk is higher in patients who have been exposed to certain drugs, including digoxin, phenytoin, and chemotherapeutic agents. These lymphomas may also contain identical Epstein - Barr virus (EBV) episomes, a finding that indicates that the tumour originated from a single EBV-infected cell [7].

The most common sites of extranodal NHL are the gastrointestinal tract [8] and the head and neck region [2]. In the head and neck sites, NHLs have been reported, in order of decreasing frequency, in the Waldeyer ring, tonsils, tongue base, nose/paranasal sinuses, orbit, and salivary glands [2]. In the oral cavity, the most common sites of NHL are the gingiva and hard palate. Other intraoral sites include the buccal mucosa, tongue, floor of the mouth, and lips.

Both patients were noted to have lesion of the tongue, but tongue lesions are noted in the literature to be extremely rare [[1], [2], [3]]. When tongue lymphomas do occur, most are of B-cell origin; the diffuse large-cell variety is the most common. Extranodal lymphomas of the T cell phenotype tend more to be sinonasal in origin than of the tongue [2], with T cell lymphomas of the tongue being even rarer than B cell lymphomas [9]. Our first patient’s tumour was of the T-cell type, while our second patient had a diffuse large B cell lymphoma both of which were confirmed by immunohistochemistry. Immunohistochemistry helped not only in making the diagnosis, but also in classifying these lesions. Immunohistochemical markers for high grade T cell lymphoma & Diffuse B Cell Lymphoma is as shown in the Table 1.

**Table 1:** Showing the immune-histo-chemical stains used for high grade T cell lymphomas and Diffuse B cell lymphoma. The positive stains were indicated by the tick () and the negative stains as indicated by the cross (x).

STAINS	Precursor T-Cell Lymphoma/leukemia	Anaplastic Large Cell Lymphoma (ALCL)	Peripheral T-Cell Lymphoma, NOS	Diffused B-Cell Lymphoma
BCL6				√
CD1a	√			
CD3	√	√	√	
CD4	√	√	√	x
CD5		√	√	
CD7		√	√	
CD8	√	√	√	
CD10				√
CD15		√		
CD19				√
CD20	√	√	√	√
CD22				√
CD30		√	√	
CD34				
TdT	√			x
CD79a				√
CD99	√			
Ki67	√		√	
ALK		√		
IRF4/MUMI				√

On histology, T - cell lymphomas typically demonstrate a pleomorphic mixture of small, medium, and large malignant T - cells with a diffuse effacement of lymphoid architecture. Diffuse Large B Cell Lymphomas on the other hand, present as one of three morphological variants: centroblastic, immunoblastic and anaplastic.

Finally, differentiating a NHL from a carcinoma is critical for the treatment of these lesions, as NHLs are highly sensitive to chemotherapy [10] and in some cases radiotherapy. Low-grade localized lesions (e.g., stage I and II) can be treated with radiation alone, but intermediate-grade, high-grade, and advanced-stage NHLs require a combination of radio/chemotherapy [1,11,12].

## 5 Conclusion

With regards to tumours arising in the tongue, squamous cell carcinomas are still classified as the most common. Lymphomas however, should still be kept in consideration as a differential diagnosis with regards to lesions arising from this site. This is crucial to note, as the management and prognosis of lymphomas is different, and early detection can result in a complete cure and better long-term survival.

## Conflicts of interest

There is no conflicts of interest amongst the authors in publishing theses case series.

## Funding

No fund was received to published this article.

## Ethical approval

Ethical approval was approved from the institutional review board of San Fernando General hospital.

## Consent

Written informed consent was obtained from the patients for publication of this case report and accompanying images. A copy of the written consent is available for review by the Editor-in-Chief of this journal on request.

## Author contribution

The first author (JC), third author (SI), fourth author (CR) and the fifth author (MC) have helped in data collection, literature search as well as designing and organizing to write manuscript. The fifth author (SM) has helped in critics the entire manuscript’s design and its contents.

All authors have approved the final version of this manuscript.

## Registration of research studies

It is registered with Research registry.

The UIN is researchregistry4386.

## Guarantor

The corresponding author will accept the full responsibility for the work.

## Provenance and peer review

Not commission, externally peer reviewed.
